# R-wave peak time at lead II in Chinese healthy adults

**DOI:** 10.1186/s12872-016-0258-7

**Published:** 2016-05-10

**Authors:** Jinhua Deng, Tingting Chen, Chujuan Zeng, Nan Lu, Lihong Zhou, Xuerui Tan, Min Yu

**Affiliations:** Mianyang Central Hospital, Mianyang, Sichuan 621000 China; Department of Cardiology, the First Affiliated Hospital, Shantou University Medical College, Shantou, Guangdong 515041 China

**Keywords:** Wide QRS complex tachycardia, Electrocardiography, Supraventricular tachycardia, Ventricular tachycardia

## Abstract

**Background:**

Wide QRS complex tachycardia (WCT) is a common arrhythmia. How to differentiate between WCTs is a challenge in clinical practice. Recently R-wave peak time (RWPT) at lead II was reported to be a helpful and simple tool for differentiating WCTs. However, it has remained unknown about the reference range of RWPT at lead II. In present study, we aimed to investigate the reference range of RWPT at lead II in Chinese healthy adults.

**Methods:**

A retrospective study was conducted in the First Affiliated Hospital of Shantou University Medical College in Southern China. Two thousand four hundred healthy adults aged 21–80 years with no history of structural heart diseases were included. RWPT at lead II was determined.

**Results:**

Of 2400 healthy adults, 1200 men and 1200 women were included. The differences of age, mean heart rate and mean QRS duration at lead II between male and female were not significant. RWPT ranged from 16 to 42 ms in male while from 16 to 44 ms in female. The 95 % reference range of RWPT in normal male and female are 19.91 ~ 39.55 ms and 21.75 ~ 37.67 ms, respectively. Compared with the female, the male had a significantly longer RWPT at lead II (29.73 ± 5.01 ms vs 29.71 ± 4.06 ms in female, *P* = 0.000).

**Conclusion:**

Our study showed that RWPT at lead II is different between male and female. The male had a significantly longer RWPT at lead II than the female.

## Background

Wide QRS complex tachycardia (WCT) is a common arrhythmia, which is defined as a rhythm with a rate >100/min and a QRS duration >120 ms. Most of WCT are caused by ventricular tachycardia (VT) or supraventricular tachycardia (SVT) with abnormal intraventricular conduction (SVT-A). Therefore, the main problem in the clinic in the differential diagnosis of WCTs is to distinguish VT from SVT-A. Despite several algorithms and criteria have been proposed [1–3], making an accurate rapid differentiating between VT and SVT-A remains a significant clinical problem. The morphological criteria included in these algorithms were difficult to recall especially in an urgent setting. Recently Pava et al. [4] reported a novel method to differentiate between WCTs, with R-wave peak time (RWPT) ≥50 ms at lead II for VT diagnosis. Though the lead II RWPT method seems to be inferior to multi-step algorithms in later research [5], it is still a helpful and simple tool for differentiating VT from SVT.

However, it has remained unknown about the reference range of RWPT at lead II. In present study we aimed to investigate the RWPT at lead II in Chinese healthy adults and the effects of gender and age on RWPT.

## Methods

A retrospective descriptive study was conducted in the First Affiliated Hospital of Shantou University Medical College in Southern China. ECGs with sinus rhythm between January 2014 and October 2015 were reviewed from healthy persons aged 21–80 years with no history of structural heart diseases. ECGs with bundle branch block (BBB, including right and left bundle branch block and fascicular blocks) and ventricular hypertrophy were excluded. The healthy adults were divided into groups according to gender and age. A total of 2400 healthy adults were included. The RWPT at lead II was determined according to the method described by Pava et al. [4]. The study was approved by the ethics committee of Shantou University Medical College.

Data are presented as the number of patients/controls or mean ± SD. Differences between group means were assessed by an unpaired Student’s *t*-test for single comparisons or by ANOVA for multiple comparisons using SPSS 16.0. *P* value < 0.05 was considered significant.

## Results

In present study, there are 2400 ECGs (one ECG per person). One thousand two hundred men and 1200 women were included. The differences of age, mean heart rate and mean QRS duration at lead II between the male and female were not significant (Table 1). RWPT ranged from 16 to 42 ms in male while from 16 to 44 ms in female. The 95 % reference range of RWPT in normal male and female are 19.91 ~ 39.55 ms and 21.75 ~ 37.67 ms, respectively. Compared with the female, the male had a significantly longer RWPT at lead II than the female (29.73 ± 5.01 ms vs 29.71 ± 4.06 ms in female, *P* = 0.000). However, the differences of RWPT between the male and female in different age groups are varied. RWPT was longer in the female than in the male from age 21 to 40 while the male’s RWPT was more longer between age 71 and 80. No differences in RWPT were noted between the female and male at age 41 to 70. There is a longer RWPT at age 61 ~ 70 (30.41 ± 4.80 ms) in the male group while at age 31 ~ 40 (30.75 ± 3.40 ms) in the female group (Table 2).

**Table 1:** Summary of main study results

	Male	Female	*P* value
Number	1200	1200	
Age (years, mean ± SD)	50.27 ± 16.77	50.11 ± 16.68	0.626
Mean heart rate (bpm)	73.94 ± 9.14	75.28 ± 8.62	0.058
Mean QRS duration at lead II (ms)	90.10 ± 8.38	82.98 ± 8.21	0.997
R-wave peak time at lead II (ms)	29.73 ± 5.01	29.71 ± 4.06	0.000
Minimum (ms)	16	16	
Maximum (ms)	42	44	
95 % reference range (ms)	19.91 ~ 39.55	21.75 ~ 37.67

**Table 2:** R-wave peak time at lead II at different subgroups

Subgroup (age)	R-wave peak time at lead II (ms) male	R-wave peak time at lead II (ms) female	*P* value
21 ~ 30	28.98 ± 4.87	30.03 ± 3.54^c^	0.000
31 ~ 40	29.67 ± 4.60	30.75 ± 3.40^b c^	0.000
41 ~ 50	30.09 ± 5.08	29.35 ± 4.62	0.181
51 ~ 60	29.95 ± 4.91	29.54 ± 4.95	0.502
61 ~ 70	30.41 ± 4.80^a^	29.65 ± 5.16	0.453
71 ~ 80	29.26 ± 5.63	28.95 ± 4.64^c^	0.005

^a^Compared with group age 21 ~ 30 in the male group, ^b^compared with group age 41 ~ 80 in the female group, ^c^compared with corresponding group in the male

## Discussion

In present study we measured the duration of RWPT at lead II in 2400 Chinese healthy adults with no structural heart diseases and no BBB. We found that the 95 % reference range of RWPT in normal male and female are 19.91 ~ 39.55 ms and 21.75 ~ 37.67 ms, respectively. The male had a significantly longer RWPT at lead II than the female.

To the best of our knowledge, this is the first study investigating the reference range of RWPT at lead II in Chinese healthy adults. Normally, the cardiac stimulus starts in the sinus node and spreads through the right and left atria, the atrioventricular (AV) node and bundle of His. Finally, the cardiac stimulus spreads to the ventricular muscle cells through the Purkinje fibers. The speed of electrical impulses through different parts of the heart varies. It was reported that conduction velocity is faster in the His–Purkinje system than in ventricular myocardium [6], accordingly the QRS duration was varied in SVT and VT. The duration of RWPT at lead II was also different. That is the underlying basis of why RWPT at lead II can be used to distinguish VT from SVT-A. Recently Pava et al. [4] reported that RWPT at lead II in patients with SVT was significantly narrower than in patients with VT (26.8 ± 9.5 ms and 76.7 ± 21.7 ms). RWPT ≥50 ms at lead II had a sensitivity of 93 % and specificity of 99 % in the differential diagnosis of WCTs. However, the greater sensitivity and specificity were not verified by later researches [5, 7–9], which showed that the sensitivity and specificity ranged from 65.7 to 79.1 % and 80.9 to 97 %, respectively. Jastrzebski et al. [6] reported the accuracy for RWPT at >50 ms for VT was only 69 % and RWPT of 42.5 ms was more optimal for differentiating VT from SVT than 50 ms. These researches showed that RWPT at lead II may vary with different population. In present study, the longest RWPT was 44 ms in 2400 healthy adults, suggesting that RWPT of 50 ms may be optimal to differentiate between VT and SVT.

Another interesting thing is that RWPT at lead II is different between male and female. In the clinic, it is not uncommon that some ECG parameters have gender differences. The upper limit of the QTc (rate-corrected QT interval) is 0.43 s in men while 0.45 s in women. In the diagnosis of ST-elevation myocardial infarction (STEMI), the degree of ST segment elevation in at least two contiguous leads varies between male and female. Though it seems the very significant difference between males and females in RWPT duration (29.73 ± 5.01 ms vs 29.71 ± 4.06 ms in female, *P* = 0.000), the difference is only 0.02 ms, which is a statistic or clinical difference remained to be further determined.

A few limitations were apparent in present study. First, the population of the study is small, large sample would be needed to determine the reference range of RWPT at lead II. Secondly, owing to the geographic difference of ECG, the reference range in Chinese healthy population may be different from other regions in the world.

## Conclusions

In conclusion, we found that the reference range of RWPT at lead II in male was different from in female. The male had a significantly longer RWPT than the female. Our findings provided the basis for the use of RWPT in the differential diagnosis of WCTs.

## Availability of data and materials

Raw data supporting the obtained results are available at the corresponding author.

## Ethical approval

The study was approved by the ethics committee of Shantou University Medical College.

## Consent for publication

Not applicable.

## Abbreviations

RWPT: R-wave peak time
SVT: supraventricular tachycardia
VT: ventricular tachycardia
WCT: wide QRS complex tachycardia
